# Outcomes of Candida and Non-Candida Aortic Graft Infection

**DOI:** 10.1177/15385744221129236

**Published:** 2022-09-23

**Authors:** Safwan Omran, Steffen Gröger, Bashaer Shafei, Larissa Schawe, Leon Bruder, Haidar Haidar, Andreas Greiner

**Affiliations:** 1 Department of Vascular Surgery, Corporate Member of Freie Universität Berlin, Humboldt-Universität zu Berlin, and Berlin Institute of Health, 14903 Charité––Universitätsmedizin Berlin, Berlin, Germany

**Keywords:** Candida, fungal, graft, infection, aortic repair

## Abstract

**Objective:**

To evaluate and compare the outcomes of Candida- and non-Candida-associated aortic graft infections.

**Methods:**

We retrospectively analyzed the data from patients treated for aortic graft infection from 2015 to 2021 in our hospital.

**Results:**

A total of 66 patients (56 men; median age, 69 years; range, 50-87 years) were admitted with aortic graft infection, including 21 (32%) patients in the Candida group and 45 (68%) in the non-Candida group. The average time between initial operation and presentation of aortic graft infection was 50 months (range, 1-332 months). Graft-enteric fistulas (GEFs) were more often in the Candida group (57% vs 27%, *P* = .017). The most proven causative fungal specimen was *C. albicans* in 16 (76%) patients. Non-albicans Candida was found in 9% of all patients and 29% of the Candida patients. The median ICU length of stay was longer in the Candida group than non-Candida (10 vs 9 days, *P* = .012). Additionally, the median hospital length of stay was longer in the Candida group (33 vs 22 days, *P* = .048). There were no statistically significant differences between Candida and non-Candida groups according to the in-hospital mortality (24% vs 24%, *P* = .955), and 1-year mortality (38% vs 38%, *P* = .980).

**Conclusions:**

Patients with bacterial and fungal aortic graft infections have high rates of morbidity and mortality. We found no significant differences in postoperative morbidity and mortality between Candida and non-Candida patients. However, the ICU and hospital length of stay were longer in the Candida group.

## Introduction

Aortic graft infections (AGI) are rare and life-threatening conditions^1^ with occurrence ranging from .2 to .7% after endovascular aortic repair (EVAR) and 2% after open aortic repair.^2,3^ The microbiological cause of AGI has changed over the years.^4^ Whereas *Staphylococcus aureus* was the predominant microorganism in the early studies,^5,6^ multidrug-resistant strains, polymicrobial infection, and Candida species became increasingly the main causative pathogens for AGI.^4^

Although rare, AGI is associated with high morbidity and mortality rates between 25% and 60%.^7^ Optimal procedural management in diagnosis, surgical treatment, and the antibiotic regime, are still subject to controversy.^8^ Whereas numerous studies focused on the surgical options for the treatment of AGI, and many showed the spectrum of pathogens, only a few dealt with the effect of fungal-associated AGI (Candida) on the outcome.^9^ Existing ones mostly case reports mainly describing disastrous clinical courses.^10-13^

Therefore, our present study aimed to evaluate the impact of Candida on the outcomes after AGI surgical repair.

## Material and Methods

After approval of the Ethics Committee of the Charité – Universitätsmedizin Berlin this study (Number: EA4/017/19), we conducted a retrospective single-center study. The patient’s informed consent was waived. All consecutive patients with aortic graft infection from 2015 until 2021 in a single-center institution were included in the present study. The data were prospectively collected in a database and retrospectively analyzed for the present study.^14-16^ Preoperative blood cultures, smears of the prosthesis, sonication, periprosthetic fluids, or aneurysms were obtained and cultured with standard anaerobic and aerobic methods. Vascular graft infection (VGI) was defined as clinical and laboratory evidence of infection in which graft cultures grew microorganisms or cultures showed no bacterial or fungal growth but with a radiological and clinically confirmed graft infection. According to the isolated germs, patients were classified into Candida and non-Candida groups. The candida group was defined as VGI in which Candida was grown from the excised graft or the perivascular liquid obtained at surgery or using a CT-guided puncture. The non-Candida group was composed of VGI in which graft cultures grew other microorganisms or in which cultures remained sterile. If Candida was grown from blood culture but not from the excised graft, the patients were classified in the non-Candida group.

According to the time after the initial operation, graft infections occurring within 4 months after implantation were classified as early infections, and those occurring after 4 months as late infections.

Additionally, patients with graft-enteric fistulas (GEF) were classified according to the type of the fistula into true GEFs and paraprosthetic-enteric fistulas (PPEFs). True GEFs are communication between the aortic and the bowel lumen, and PPEFs are characterized by erosion of the bowel by an underlying prosthesis. All patients received a computed tomography (CT) scan. In the case of suspected graft infection without any proof of bacteria preoperatively, the patients underwent positron emission tomography (PET-CT).

The preoperatively collected data included patient demographics, cardiovascular risk factors, clinical presentation, imaging findings, aortic pathology and previous procedures, the reason for initial operation, laboratory findings, and isolated germs. We also calculated the interval between the initial aortic intervention and the development of an AGI. In addition, the physical status of all patients was assessed preoperatively using the American Society of Anesthesiologists classification.

The operative details that were considered included the surgical approach, type of reconstruction, use of aortic cross-clamping, used revascularisation graft, and operative time. Every patient, who needed an elective operation, underwent a multi-disciplinary discussion with vascular surgeons, radiologists, and anesthetists to choose between the available surgical options. On the other hand, the decision-making in urgent situations was dependent on the surgeon’s preference and the availability of reconstruction material in most cases.

The factors considered for choosing the surgical approach included the severity of the sepsis, the urgency of the operation, the availability of cryopreserved human allografts or deep femoral vein, and the patient’s comorbidities. The patients either received an in-situ-reconstruction (ISR) or an extra-anatomic reconstruction (EAR). However, ISR was the preferred technique whenever it was possible. Independently of the type of reconstruction, the infected and avital tissue was radically resected. ISR was done by replacing the removed graft with homograft, deep femoral vein, silver prosthesis, rifampicin soaked dacron prosthesis, or constructing a pericardial tube. Homograft or deep femoral veins were preferred if available. All ISR operations were done via a midline laparotomy.

On the other hand, EAR included one of the following variations: bilateral axillofemoral bypass grafts and one-side axillofemoral with crossover bypass. The bypass grafts were completed with 8 mm polytetrafluoroethylene in all cases. Laparotomy was subsequently performed to remove the infected aortic graft and close the aortic stump.

Antibiotic therapy was empirically initiated right after sampling and adjusted concerning the antibiogram-resistogram. Postoperative intravenous antibiotics during the hospital stay and supportive oral antibiotics for at least 3 months were administrated for all patients. All Candida patients received antifungal treatment with oral fluconazole (400 mg x 1) or intravenous caspofungin (50 mg x 1) during the hospital stay and then fluconazole (50 mg x 2) for at least 3 months.

The primary endpoint was the 30-day, 180-day, and 365-day mortality independently from its cause. Secondary outcomes include the occurrence of any complication, length of stay in ICU, and hospitalization.

Additionally, we made uni- and multivariate analysis of the variables to predict the 30-day in-hospital mortality. The variables included cardiovascular risk factors, gender, GEF, candida infection, ISR, EAR, supra-celiac clamping, and EVAR infection.

### Statistical Analysis

As a descriptive study, the data were evaluated individually. Moreover, continuous variables were expressed as mean (SD). Data without distributed data are expressed by the median and interquartile range (IQR). Proportional data is constituted as a percentage as well as the number of patients. The chi-square test was used for differences between the groups of non-continuous variables, whereas students' unpaired t-test was used for metric variables. For survival analysis, Kaplan Meier was used. A multivariate model (binary logistic regression) in a stepwise fashion was constructed to identify factors independently associated with higher 30-day mortality rates. Variables found to predict 30-day in-hospital mortality on univariate analysis at a *P*-value of < .2 were entered into the multivariate analysis. Models were tested for goodness of fit using the Hosmer-Lemeshow statistic.

Statistical significance was considered as a *P*-value of < .05. SPSS Statistics 26 (IBM, Armonk, NY, USA) was used for statistical analysis.

## Results

### Patients

From February 2015 to December 2021, a total of 691 abdominal aortic repairs, including 390 OARs and 301 EVARs, were performed at our institution. Of the 691 patients, 66 (56 males, mean age 69 ± 12 years) were admitted with aortic graft infection, accounting for 9.6% of all abdominal aortic repairs at our hospital. Of the 66 patients, 23 (35%) patients had undergone the initial surgery at our clinic, and 43 (65%) had undergone the initial surgery at an external hospital.

### Diagnostics and Clinical Presentation

According to the bacteriologic specimens obtained from culture studies from the excised graft or the perivascular liquid, 21 (32%) patients were classified in the Candida group and 45 (68%) in the non-Candida group. Four patients had had candidemia with Candida grown from blood culture but not from the graft. These patients were classified in the non-Candida group. The patient’s comorbidities, clinical signs, and laboratory findings are listed in Table 1. Both groups had similar risk factor profiles. The clinical presentation was related to the location of the infection and the presence and type of aorto-duodenal fistula. The symptoms and signs included fever in 42 (64%) patients, abdominal pain in 29 (44%), hemorrhagic shock in 12 (18%), and psoas abscess in 10 (16%) patients. Additionally, 6 (9%) patients underwent cardiopulmonary resuscitation at presentation due to hemorrhagic shock by aorto-duodenal fistulas.

**Table1.** Descriptive Characteristics and Clinical Presentation.

Characteristics	Total	Candida	Non-Candida	*P*
Number	66 (100)	21 (32)	45 (68)	
Demographics				
Age -years	69 (50-87)	72 (57-79)	69 (50-87)	.615
Male	56 (85)	18 (86)	38 (84)	.893
Risk factors				
Coronary artery disease	36 (55)	14 (67)	22 (49)	.177
Diabetes mellitus	17 (26)	5 (24)	12 (27)	.805
Hypertension	57 (86)	20 (95)	37 (82)	.252
Hyperlipoproteinemia	21 (32)	7 (33)	14 (31)	.857
Stroke	6 (9)	2 (10)	4 (9)	1
COPD	23 (35)	7 (33)	16 (36)	.860
Chronic renal insufficiency	25 (38)	9 (43)	16 (36)	.569
Smoking	27 (41)	7 (33)	20 (44)	.392
Peripheral vascular disease	29 (44)	7 (33)	22 (49)	.236
Malignancy	13 (20)	4 (19)	9 (20)	1
ASA class				
II	2 (3)	1 (5)	1 (2)	
III	47 (71)	15 (71)	32 (71)	
IV	17 (26)	5 (24)	12 (27)	
Initial pathology				
Aortic aneurysm	42 (64)	14 (67)	28 (62)	.727
PAD	24 (36)	7 (33)	17 (38)	.727
Initial aortic repair				
Endovascular	18 (28)	5 (25)	13 (29)	.666
Open repair	47 (72)	16 (76)	32 (71)	.666
Etiology				
Graft-enteric fistulas	24 (36)	12 (57)	12 (27)	.017*
Aortic graft infection	42 (64)	9 (43)	33 (73)	.017*
Clinical presentation				
Fever	42 (64)	17 (81)	25 (56)	.046*
Abdominal pain	29 (44)	12 (57)	17 (38)	.140
Hemorrhagic shock	12 (18)	5 (24)	7 (16)	.499
Psoas abscess	10 (16)	2 (11)	8 (19)	.710
Hematemesis	9 (14)	2 (10)	7 (16)	.506
Resuscitation	6 (9)	2 (10)	4 (9)	1
Septic shock	6 (9)	0	6 (13)	.166

CPR: C-Reactive Protein; EVAR, endovascular aortic repair; OAR, open aortic repair; PPEF, paraprosthetic–enteric fistula; PAD, peripheral aortic disease.

We found similar laboratory findings between both groups in the laboratory examination at admission. Esophagogastroduodenoscopy has been performed in 27 (41%) patients with suspicious aorto-duodenal fistula. However, the aorto-duodenal fistula was diagnosed in only 3 cases based on esophagogastroduodenoscopy. All patients underwent CT-angiography at presentation. The diagnostic CT findings included a periprosthetic fluid collection in 49 patients (74%), trapped air in 22 (33%), and psoas abscess in 10 (15%). Additionally, 5 (9%) patients underwent fluorine-18-fluorodeoxyglucose positron emission tomography, showing signs of graft infections.

### Pathology and Etiology

The initial reason for aortic surgery was abdominal aortic aneurysm in 42 (64%) patients and peripheral arterial disease in 24 (36%) patients. An endovascular procedure was done in 18 (28%) patients and open aortic repair in 47 (72%) patients. The average time between initial operation and presentation of aortic graft infection was 50 months (range, 1-332 months), with no difference between Candida (52 months, range 1-139 months) and non-Cndida (50 months, range 1-332 months) groups with *P* = .921. Early infections (<4 months after initial surgery) was noted in 10 (15%) patients and late infections (>4 months after initial surgery) in 56 (85%) patients. GEFs were found in 24 (36%) patients and were more often in the Candida group (57% vs 27%, *P* = .017).

### Bacteriology

Blood cultures were obtained in all patients, and cultures from the aneurysmal content and the arterial wall in 63 (96%) patients. CT-guided puncture of the peri-graft fluid was obtained in 3 patients. The results of the cultures are summarized in Table 2. Sonication of the vascular grafts was obtained in 18 (27%) patients. Of those, 11 were positive, and 7 were negative.

**Table 2.** Blood and Intraoperative Specimen Cultures Results.

	Candida N = 21	Non-Candida N = 45	Total N = 66
Blood cultures			
Positive	11 (52)	23 (51)	34 (52)
*Escherichia coli*	3 (14)	5 (11)	8 (12)
*Staphylococcus* spp	2 (10)	14 (31)	16 (24)
*Streptococcus* spp	1 (5)	2 (4)	3 (5)
*Klebsiella pneumoniae*	1 (5)	1 (2)	2 (3)
*Bacteroides fragilis*	1 (5)	1 (2)	2 (3)
*Enterococcus faecium*	2 (10)	4 (9)	6 (9)
Candida	4 (19)	4 (9)	8 (12)
Graft/perigraft smears			
Positive	21 (100)	24 (53)	45 (76)
*Escherichia coli*	4 (19)	5 (11)	9 (15)
*Staphylococcus* spp	2 (10)	12 (28)	14 (22)
*Streptococcus* spp	3 (14)	4 (9)	7 (12)
*Klebsiella pneumoniae*	2 (10)	3 (7)	5 (8)
*Bacteroides fragilis*	0	2 (5)	2 (3)
*Enterococcus faecium*	7 (33)	2 (5)	9 (15)
*Enterococcus faecalis*	2 (10)	3 (7)	5 (8)
Candida	21 (100)	0	21 (36)

Blood culture was positive in 34 (52%) of the patients. The most common fungal and bacterial specimen in the blood culture were *Staphylococcus* spp (n = 16, 24%), *E. coli* (n = 8, 12%), Candida (n = 8, 12%), and *Enterococcus faecium* (n = 6, 9%). Culture studies from the excised graft or the perivascular liquid were positive in all patients in the Candida group and 53% of the patients in the non-Candida group. The most common fungal and bacterial specimen was Candida (n = 21, 36%), *Staphylococcus* spp (n = 14, 22%), *E. coli* (n = 9, 15%), and *Enterococcus faecium* (n = 9, 15%).

Candida was found in 21 (32%) of the excised graft. Whereas 18 (86%) of the Candida patients had had one specie, two patients developed two species and one 3 species. The most proven causative specimen was *C. albicans* in 16 (76%) patients. Non-albicans Candida was found in 9% of all patients and 29% of the Candida patients. The most frequently non-albicans Candida was *Candida glabrata* in 4 (19%) patients, followed by *C. parapsilosis* in 2 (10%), *C. tropicalis*, C. kefyr, and *C. dubliniensis* in one (5%) patient for each (see Table 3). According to the time of infection, no statistically significant difference was found between patients with early and late infection in candida infections (24% vs 11%, *P* = .180).

**Table 3.** Candida Species.

	Candida N = 21
Number of candida species pro patients	
One	18 (86)
Two	2 (10)
Three	1 (5)
Candida species	
*C. albicans*	16 (76)
*C. glabrata*	4 (19)
*C. tropicalis*	1 (5)
*C. parapsilosis*	2 (10)
*C. kefyr*	1 (5)
*C. dubliniensis*	1 (5)

### Operative Details

**Figure 1.**
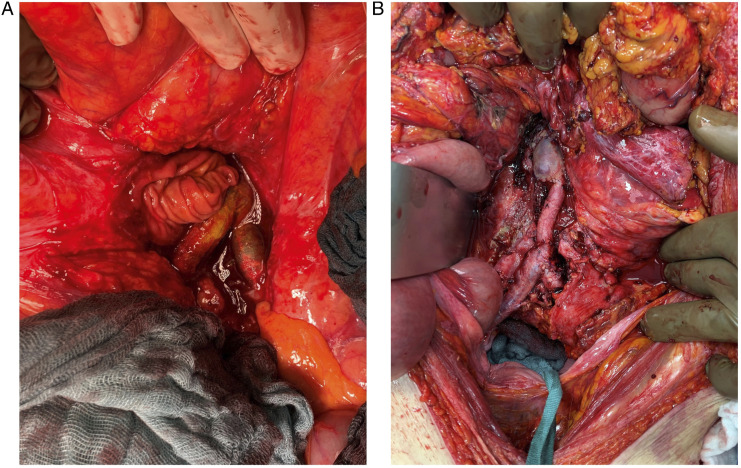
78-year-old man presented with abdominal pain and signs of infection. Blood culture grew *Escherichia coli* and *Serratia marcescens*. The culture of the excised graft grew *Candida glabrata*, *Candida dubliniensis*, *Enterococcus faecium*, and *Staphylococcus haemolyticus*. (A) intraoperative imaging showing graft-enteric fistula. (B) Intraoperative imaging shows aortobifemoral bypass using the femoral veins.

The details of the treatment and outcomes are listed in Table 4. According to the surgical treatment, ISR was performed in 48 (73%) patients and EAR in 13 (20%). Additionally, 1 (1.5%) patient underwent removal of the infected graft without reconstruction, and 4 (6%) underwent conservative treatment because of the high surgical risk. ISR procedures included neoaortoiliac system (NAIS) in 21 (32%), cryopreserved homograft in 11 (17%), rifampicin-soaked graft in 11 (17%), silver-coated graft in 6 (9%), and bovine pericardial patch in 2 (3%) see Figure 1. On the other hand, EAR procedures included bilateral axillofemoral bypass grafts in 10 (15%) patients, and axillofemoral with femorofemoral bypass in 3 (5%) patients.

**Table 4.** Details of the Surgical Treatment, Complications, and Outcome.

Variables	Total N = 66	Candida N = 21	Non-Candida N = 45	*P*
Type of therapy				
ISR	48 (73)	14 (67)	34 (76)	
EAR	13 (20)	7 (33)	6 (13)	
No reconstruction	1 (1.5)	0	1 (2)	
Conservative therapy	4 (6)	0	4 (9)	
ISR				
Rifampicin-soaked graft	9 (14)	1 (5)	8 (18)	
Neoaortoiliac system (NAIS)	21 (32)	9 (43)	12 (27)	
Homograft	11 (17)	2 (10)	9 (20)	
Silver-coated dacron graft	5 (8)	2 (10)	3 (7)	
Bovine pericardial patch	2 (3)	0	2 (4)	
EAR				
Bilateral axillofemoral bypass	10 (15)	6 (29)	4 (9)	
Axillofemoral + fem-fem bypass	3 (5)	1 (5)	2 (4)	
Aortic clamping				
Infrarenal	38 (58)	14 (67)	24 (53)	
Suprarenal	5 (8)	2 (10)	3 (7)	
Supraceliac	19 (29)	5 (24)	14 (31)	
Complications				
Cardiac complications	18 (27)	5 (24)	13 (29)	.666
Acute renal failure	34 (52)	16 (76)	18 (40)	.006*
Respiratory failure	41 (62)	13 (62)	28 (62)	.980
Cerebrovascular accident	4 (6)	2 (10)	2 (4)	.587
Graft disruption	3 (5)	1 (5)	2 (4)	1
Colonic ischemia	16 (24)	7 (33)	9 (20)	.239
Small intestine infarction	12 (18)	6 (29)	6 (13)	.175
Limb ischemia	19 (29)	9 (43)	10 (22)	.085
Inguinal infection	21 (32)	8 (38)	13 (29)	.455
Multiple organ failure	16 (24)	6 (29)	10 (22)	.575
Major amputations	7 (11)	1 (5)	6 (13)	.416
Outcome				
ICU (days)	10 (0-71)	17 (3-71)	9 (0-53)	.012*
Hospital stay-days	27 (1-109)	33 (4-80)	22 (1-75)	.048*
30-Mortality	16 (24)	5 (24)	11 (24)	.955
6-months mortality	25 (38)	8 (38)	17 (38)	.980
One-year mortality	25 (38)	8 (38)	17 (38)	.980
Overall mortality	30 (46)	12 (57)	18 (40)	.193

ISR, In situ reconstruction; EAR, Extra-anatomic reconstruction; fem-fem bypass, femorofemoral crossover bypass.

The median duration of operation was 420 minutes (range, 173-846 minutes) with no statistically significant difference between Candida and non- Candida groups. Infrarenal clamping was obtained in 38 (58%) patients, suprarenal clamping in 5 (8%), and supraceliac clamping in 19 (29%).

### Postoperative Course and Outcomes

All but two patients were admitted to the ICU after the operation. The ICU length of stay was longer in the Candida group (median 10 days, range 3-71 days) than in the non-Candida group (median 9 days, range 0-53 days) with (*P* = .012). Additionally, the hospital length of stay was longer in the Candida group (median 33 days, range 4-80 days) than in the non- Candida group (median 22 days, range 1-75 days) with (*P* = .048).

Postoperative complications occurred in 58 (95%) patients. There were no statistically significant differences between Candida and non-Candida according to the postoperative complications except for acute renal failure (76% vs 40%, *P* = .006) see Table 4.

**Figure 2.**
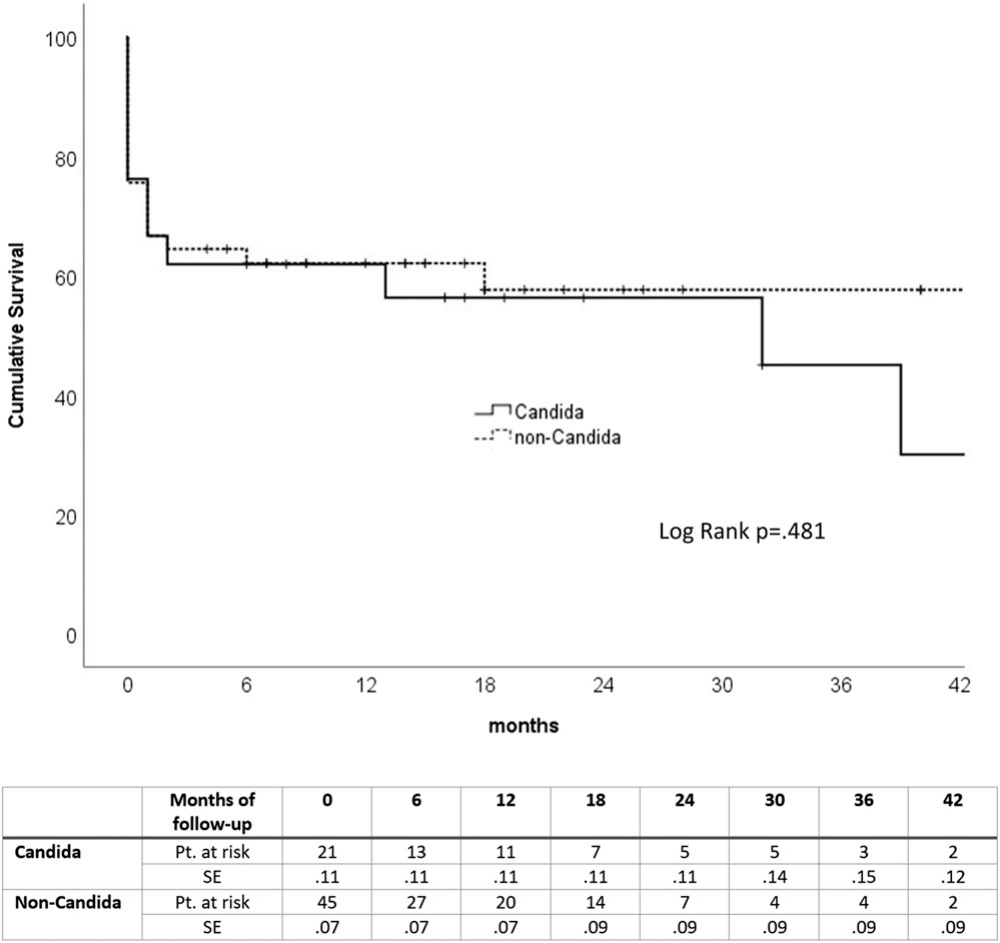
Kaplan-Meier survival estimates of the patients treated for Candida and non-Candida aortic graft infections (Kaplan-Meier log-rank test, *P* = .481). The mean survival time of candida patients was 28 months (95% CI: 16.3-40.2), and for non-candida patients, 47 months (95% CI: 35.3-59.0). SE, standard error; Pt. at risk, Patients number at risk.

There were no statistically significant differences between Candida and non-Candida groups according to the in-hospital mortality (24% vs 24%, *P* = .955), 1-year mortality (38% vs 38%, *P* = .980), and overall-mortality during the follow-up (57% vs 40%, *P* = .193) see Figure 2. Additionally, the in-hospital mortality of the patients with non-albican Candida was 50% vs 13% of those with *Candida albicans*, and the 6-months mortality of the patients with non-albican Candida was 67% vs 27% of those with *Candida albicans*.

Based on the isolated strains, postoperative intravenous antibiotics and antifungals were administered to our patients for at least 6 weeks. After that, the administration of oral suppressive antibiotics depended on the clinical and laboratory parameters of infection.

The variables with *P*-value < .2 in the univariate analysis of the 30-day in-hospital mortality included age >64 years (OR 1.4, 95% CI: 1.2-1.7, *P* = .015), ASA class 4 (OR 4.6, 95% CI: 1.3-15.4, *P* = .011), GEF (OR 3.0, 95% CI: .9-9.6, *P* = .057), and EAR (OR 4.4, 95% CI: 1.2-16.5, *P* = .056). The multivariate analysis revealed age >64 years (*P* = .001), ASA class 4 (*P* < .001), and EAR (*P* = .023) significantly associated with higher 30-day mortality. The Hosmer-Lemeshow goodness-of-fit test statistic (chi-square) was 4.4 with a *P*-value of .729. Candida infection revealed no statistically significant association with 30-day mortality.

## Discussion

In this single-center retrospective study, we analyzed the risk factors and outcomes of 66 patients with Candida and non-Candida aortic graft infections. We found that the study cohort groups had similar demographics, risk factors, and pre-existing morbidities except for statistically significant higher rates of chronic renal insufficiency in the Candida group. Moreover, patients in the Candida group had significantly longer ICU and hospital stays than non-candida patients. Although some studies revealed higher mortality rates in the patients with Candida aortic graft infections,^9^ the in-hospital, 6-months, and 1-year mortality rates in the current study revealed no significant differences between the studied groups.

During the study period, 9.6% of all abdominal aortic repairs at our hospital were operated on for aortic graft infections. The incidence of AGI at our hospital seems to be high compared to other studies.^2,3^ However, 35% of patients had undergone the initial surgery at our clinic, and 65% at an external hospital who were referred to our tertiary referral center.

According to the Candida species, *Candida albicans* was the most commonly reported species in our study and the literature.^11,17^ Additionally, patients with non-albicans Candida were more virulent than *Candida albicans* and caused more in-hospital and 6-months mortalities.

The 1-year mortality rate of 38% in our cohort is high but comparative to the current literature.^8,18^ Most current studies about aortic graft infections focus on the type of surgical reconstruction and its impact on clinical outcomes.^2,7,19-21^ However, only a few studies focused on the outcomes of the surgical treatment according to bacteriology.^11^

In the current study, we used in-situ or extra-anatomic reconstructions in all patients. In the case of graft-enteric fistulas, initial EVAR may be considered a bridge therapy to stop the bleeding and control the infection before performing the open surgery.^22^ However, initial EVAR was not applied in any of our cases.

Aortic graft infections with Candida have been deemed rare and are supposed to appear more often in critically ill and immunosuppressed patients.^23^ However, fungal aortic graft infections are increasingly observed in clinical practice but have not been reported extensively. In the current study, only smears from the excised graft or the perivascular liquid were considered for the classification into Candida and non-Candida groups. However, it is controversial for patients with Candida grown in blood culture but not from the excised graft to classify in the Candida group. Therefore, our study assumed that candidemia was not a sure proof of AGI with Candida, so they were classified in the non-Candida group.

The mean interval from the index procedure to presentation with an AGI in our cohort was 50 months and was comparable with other studies.^1^ However, Semds et al found that the average time between the initial endograft placement and the infection was 22 months.^24^ Therefore, it must be assumed that the infection in most of our patients was not related to the initial procedure. Furthermore, as Candida is prone to occur in elderly and immune-deficient patients, it is even more surprising that there were no significant differences in Candida and non-Candida patients.

Similar to other studies,^25-27^ the frequency of Candida was significantly higher in the cohort with a GEF than in patients with aortic graft infections without a GEF. In addition, we found similar results in the Candida and non-Candida groups according to the complication and mortality rates. These results are contrary to those found in the study of Dorpmans et al which found worse survival rates in Candida patients.^9^ However, the ICU and hospital stays were significantly longer in Candida patients in the current study.

### Limitations

This study has some limitations, including its single-institution retrospective design and the relatively small number of patients. Additionally, the patients in which Candida was grown from blood culture but not from the excised graft were classified in the non-Candida group. However, this may be a source of controversy. Moreover, aortic graft infections are multifactorial and can be attributed to patient factors and operative characteristics.

## Conclusions

Patients suffering from Candida have comparable complication and mortality rates as non-Candida patients but seem to have prolonged recovery time.
